# Comparison of the Reproductive Outcome Between 2 and 4 mg Daily Doses of Estradiol After Hysteroscopic Adhesiolysis: A Propensity Score Matching Analysis–Retrospective Cohort Study

**DOI:** 10.3389/fendo.2022.775755

**Published:** 2022-06-13

**Authors:** Dan-Er Qiu, Wan-Lin Zhang, Jin Liu, Fang Yang, Ye Miao, Ya-Fei Tong, Xi-Feng Xiao, Xiao-Hong Wang

**Affiliations:** Reproductive Medical Center, Department of Obstetrics and Gynecology, Tangdu Hospital, Air Force Military Medical University, Xi’an, China

**Keywords:** intrauterine adhesions, hysteroscopy, reproduction, propensity score matching, estradiol

## Abstract

**Objective:**

To investigate the effect of two postoperative doses of estradiol valerate (2 and 4 mg/day) on reproductive outcomes in patients with moderate to severe intrauterine adhesions (IUAs).

**Methods:**

A retrospective cohort study was conducted at a single tertiary reproductive medical center between January 2018 and December 2019 to compare the reproductive outcomes of two doses of estradiol valerate (2 and 4 mg daily) after hysteroscopic adhesiolysis. All patients received adjuvant postoperative treatment with a Foley catheter, hyaluronic acid gel, and medication therapy. Hysteroscopy was repeated every 7 days after surgery. Multivariate regression analysis and propensity score matching (PSM) were performed to minimize intrinsic bias.

**Results:**

A total of 212 patients with moderate to severe IUAs were included: 74 patients received 2 mg of estradiol valerate daily and 138 patients received 4 mg of estradiol daily postoperatively. No significant differences were found in the reproductive outcomes between the two groups, including clinical pregnancy rates. The multivariable regression analyses both before and after PSM also showed that there was no significant difference in the menstrual improvement and clinical pregnancy rates between the two groups.

**Conclusions:**

We suggest the use of a lower dose (2 mg/day) of estradiol valerate as an adjuvant therapy for IUAs to minimize estrogen-related side effects.

## Introduction

Intrauterine adhesions (IUAs) refer to the formation of fibrous tissue adhesion in the uterine cavity, which is caused by trauma to the basal layer of the endometrium. Morphological changes in IUAs are commonly caused by a reduction in volume and deformation of the uterine cavity. Moderate to severe IUAs have significant adverse effects on women’s physical and mental health, such as hypomenorrhea, amenorrhea, infertility, and poor reproductive outcomes. Hysteroscopic adhesiolysis is the most commonly used treatment for IUAs. Although several adjuvant measures are applied to improve clinical outcomes, such as Foley catheter, intrauterine balloon, hyaluronic acid gel, hormonal therapy, stem cell therapy, or amnion graft implantation, the recurrence rate after surgery is high (1, 2) and the reproductive outcome remains unsatisfactory (3, 4).

Previous studies confirmed that the addition of exogenous estradiol is helpful in the process of surgical trauma epithelialization within the uterine cavity (5, 6). Nevertheless, given the lack of evidence regarding the effects of different doses of estradiol on the prognosis of IUAs, there is no consensus on the proper dose of estradiol after hysteroscopic adhesiolysis. In the existing literature, the estradiol dose varied widely from 2 mg of estradiol valerate daily or its equivalent (0.625 mg conjugated equine estrogen) to 12 mg of estradiol valerate daily in the existing literature (1, 7–11). Studies have indicated that higher doses of estradiol valerate [e.g., 2 (12), 4 (1, 2), and 8 mg (13)] can promote hyperplasia of the endometrium and prevent the transformation of the endometrium to the secretory phase. This is likely due to the maintenance of a high expression of estrogen receptors (ERs) in the proliferative phase of the endometrium, which benefits the migration of the endometrium to the trauma site and prevents the recurrence of IUAs.

However, in clinical observation, a high dose of estradiol valerate added after adhesiolysis failed to show significantly better uterine recovery (8, 10). This phenomenon is in accordance with some animal experiments, which have found that a high dose of estradiol can enhance the process of endometrial fibrosis (14–16). Studies by Chen et al. have shown that supraphysiological doses of estradiol could increase the production of some adhesion-promoting factors, such as transforming growth factor-β (TGF-β) and basic fibroblast growth factor (bFGF), which were hypothesized to result in fibrosis aggravation (15). However, a study revealed that the physiological dose of estradiol is more beneficial for the repair of endometrial injury (17).

Previous studies compared the efficacy of 2 vs. 6 mg and 4 vs. 10 mg daily doses of estradiol valerate in postoperative re-adhesion prevention (8, 10). Both studies did not show any benefit of higher doses of estradiol valerate on the reproductive outcomes; therefore, the researchers consistently preferred the lower dose of estradiol that was administered after surgery, as the lower dose of estradiol resulted in fewer side effects.

Nonetheless, no studies have compared the efficacy of 2 and 4 mg daily estradiol as adjuvant treatments for IUAs. In the present study, we compared the effects of two different doses of estradiol valerate (2 vs. 4 mg/day) as a postoperative adjuvant therapy on reproductive outcome to provide evidence on the proper dose of estradiol valerate and, thus, maximize the positive effects and minimize the side effects of estradiol.

## Materials and Methods

This retrospective cohort study screened all patients from the electronic medical record database of a single tertiary reproductive medical center from January 2018 to December 2019. Patients with moderate to severe IUAs who underwent hysteroscopic adhesiolysis were identified for further selection according to the inclusion and exclusion criteria. A total of 212 patients were included in the final analysis.

This study was approved by the Medical Ethics Committee of the Second Affiliated Hospital (Tangdu Hospital) of the Air Force Military Medical University. Data were anonymous; therefore, the requirement for informed consent was waived.

The inclusion criteria included the following:

1. Patients were diagnosed with moderate or severe IUAs according to the scoring system proposed by the American Fertility Society (18),
2. Patients underwent hysteroscopic adhesiolysis,
3. Patients received estradiol valerate 2 or 4 mg/day after surgery for 3–4 months, and
4. Patients were trying to get pregnant after treatment.

The exclusion criteria included the following:

1. Patients with incomplete medical records and
2. Patients complicated with any uterine malformation (including rudimentary uterine horn, unicornuate uterus, and bicornuate uterus) or severe endometritis.

### Definition of Outcomes

The reproductive outcome was the primary outcome of this study and included the following:

1. Clinical pregnancy, including ongoing pregnancy and live births. Ongoing pregnancy was defined as the continuation of intrauterine pregnancy beyond 12 weeks of gestation but not yet live birth at the end of data collection. Live birth was defined as the birth of a live baby exceeding 24 weeks of gestation.
2. A status of not yet pregnant, including no pregnancy after surgery and spontaneous abortion at the time of data collection.

Menstrual pattern after surgery was the secondary outcome of this study and included improved menstrual volume and menstrual volume that did not improve at all or even reduced after surgery. All the outcomes were obtained by a co-author (D-EQ) at the end of the 15-month follow-up period (12 months after attempts to conceive) through telephone.

All patients underwent surgery 3–7 days after menstruation ceased. Patients with amenorrhea received hormone replacement therapy before surgery. All patients underwent hysteroscopic surgery under strengthened local anesthesia (lidocaine combined with dezocine). The lithotomy position was adopted, and the operation was performed after skin preparation and draping. A hysteroscopic-guided needle bipolar electrode (Storz, Germany) was used for the surgery. The electrode was slowly placed into the uterine cavity with pulsive perfusion by normal saline, and adhesion bands were separated and removed, with attention to protect normal endometrial tissues during the operation. All procedures were performed by experienced medical teams under hysteroscopic supervision. Except for some patients who complained of tolerable pain during the surgery, there were no intraoperative or short-term complications, such as massive hemorrhage, uterine perforation, and hyperhydration syndrome, that occurred during the treatment.

A Foley catheter was placed in all patients immediately after surgery, and 5 ml of hyaluronic acid gel (YiShuKang^®^; Materia Medica Co., Changzhou, China) was infused into the intrauterine cavity through the catheter immediately. The catheter was retained in the uterine cavity along with antibiotics to prevent postoperative infection (ornidazole, 500 mg per 12 h, intravenous drip after surgery; cefazolin sodium, 1.0 g per 12 h, intravenous drip) for 5–7 days. All patients received hormone therapy from the first day after surgery, which consisted of a 2- or 4-mg daily dose of oral estradiol valerate (Progynova, Bayer, Germany) for 17–21 days, with a 20-mg daily dose of progesterone (Duphaston, Abbott, The Netherlands) for the last 7 days of estradiol therapy. In our center, the prescribed dose of exogenous estradiol was based on the serum estradiol level measured 1 day before surgery. Under physiological conditions, the peak serum estradiol level within a menstrual cycle reaches 200–300 pg/ml, which appears approximately 24 h before ovulation. Moreover, endometrial proliferation reaches its optimal state. Based on the pharmacokinetics of estradiol valerate, 2 mg of estradiol valerate taken orally resulted in the rise in estradiol concentration by 30–50 pg/ml in the serum 6–10 h after taking the drug. For example, if the preoperative serum estradiol level was 50 pg/ml, we would immediately give 4 mg of estradiol valerate (which might increase the estradiol concentration by 100 pg/ml in the serum) after surgery. The patients were treated for 3–4 cycles according to the recovery of the uterine cavity. In addition, to improve blood flow in the endometrium, aspirin tablets (0.1 g daily) were prescribed. It has been reported that new adhesions might be formed several days after surgery and an early second-look hysteroscopy is conducive to discover and remove new adhesions promptly. In addition, the recovery of the uterine cavity is a dynamic and progressive process. Multiple hysteroscopies will help physicians in evaluating the recovery of the uterine cavity dynamically and in proposing appropriate treatment strategies for patients promptly. Therefore, in our center, postoperative hysteroscopy was performed weekly, and the entire examination cycle was sustained until the uterine cavity returned to normal, or the doctor supposed that the cavity was qualified for pregnancy or a second surgical procedure was required.

Clinical parameters were extracted from the hospital’s electronic medical record system, including age, body mass index (BMI), IUA diagnosis classification (moderate or severe), preoperative menstrual pattern (regular or irregular menstrual cycle), preoperative estradiol level (estradiol < 50 pg/ml indicates the early follicular stage, 50–200 pg/ml indicates the middle follicular stage, and estradiol > 200 pg/ml indicates the late follicular stage), history of hysteroscopic surgery, number of pregnancy-related dilatation and curettage procedures, estradiol treatment regimen, pregnancy method [spontaneous pregnancy or assisted reproductive technology (ART)], and infertility factors other than IUAs [none, combined with fallopian tubal factor, combined with anovulatory factor, combined with male infertility factor, combined with complex infertility factors (two or more infertility factors)].

### Statistical Analysis

Continuous variables were presented as mean ± standard deviation (normal distribution) or median (quartile) (skewed distribution), while categorical variables were expressed as numbers and proportions. The Student’s *t*-test (normal distribution), the Wilcoxon signed-rank test (skewed distribution), and the chi-squared test (classification variable) were applied for the primary comparison between the two groups. Multivariate logistic regression analysis was performed to assess the independent effects of daily doses of estradiol valerate on reproductive outcomes. Diagnosis classification, preoperative estradiol level, age, BMI, history of hysteroscopic surgery, preoperative menstrual pattern, estradiol treatment regimen, and number of pregnancy-related dilatation and curettage procedures were adjusted as confounding factors in the adjusted models. Crude odds ratios (ORs) and adjusted ORs with 95% confidence intervals (CIs) were calculated. Moreover, propensity score matching (PSM) was conducted as a sensitivity analysis to confirm the results. EmpowerStats statistical software (http://www.empowerstats.com/en/) was used for the statistical analysis.

## Results

A total of 288 patients underwent hysteroscopic adhesiolysis during the study period. Of these, 76 (26.40%) patients were excluded for the following reasons: incomplete medical records (40 patients, 13.89%), mild IUAs (1 patient, 0.35%), uterine arterial embolization (1 patient, 0.35%), and other daily doses of postoperative estradiol valerate (34 patients, 11.81%). The remaining 212 patients were eligible for analysis, of which 74 patients took 2 mg/day of estradiol valerate and 138 patients received 4 mg/day of estradiol valerate. All demographic parameters of this cohort are presented in **Table 1**.

**Table 1:** Characteristics of the study population.

Daily dose of postoperative estradiol valerate (mg)	2 mg/day	4 mg/day	*P*
*N* (%)	74 (34.9)	138 (65.1)	
BMI (kg/m^2^)	22.32 ± 2.65	22.98 ± 3.17	0.128
Age (years)	34.96 ± 5.22	33.67 ± 4.19	0.051
Preoperative estradiol level (pg/ml)	159.24 ± 92.84	97.87 ± 94.34	<0.001
History of hysteroscopic surgery			0.547
No (%)	60 (81.1)	107 (77.5)	
Yes (%)	14 (18.9)	31 (22.5)	
Diagnosis classification			<0.001
Moderate (%)	57 (77.0)	74 (53.6)	
Severe (%)	17 (23.0)	64 (46.4)	
Preoperative menstrual pattern			0.543
Regular (%)	65 (87.8)	117 (84.8)	
Irregular (%)	9 (12.2)	21 (15.2)	
Estradiol treatment regimen			0.567
Cyclical (%)	26 (35.1)	54 (39.1)	
Continuous (%)	48 (64.9)	84 (60.9)	
Number of pregnancy-related dilatation and curettage procedures			0.283
None (%)	13 (17.6)	23 (16.7)	
One (%)	24 (32.4)	43 (31.2)	
Two (%)	17 (23.0)	47 (34.1)	
Three or more (%)	20 (27.0)	25 (18.1)	
Preoperative estradiol level			<0.001
Early follicular stage (%)	9 (12.2)	47 (34.1)	
Middle follicular stage (%)	44 (59.5)	76 (55.1)	
Late follicular stage (%)	21 (28.4)	15 (10.9)	
Pregnancy method			0.050
Spontaneous pregnancy (%)	24 (32.4)	64 (46.4)	
Assisted reproductive technology (%)	50 (67.6)	74 (53.6)	
Infertility factors other than IUAs			0.353
None (%)	40 (54.1)	90 (65.2)	
Combined with fallopian tubal factor (%)	16 (21.6)	25 (18.1)	
Combined with anovulatory factor (%)	3 (4.1)	2 (1.4)	
Combined with male infertility factor (%)	10 (13.5)	17 (12.3)	
Combined with complex infertility factors [two or more infertility factors (%)]	5 (6.8)	4 (2.9)	
Outcomes			
Menstrual pattern after surgery			0.814
Not improved (%)	32 (43.2)	62 (44.9)	
Improved (%)	42 (56.8)	76 (55.1)	
Reproductive outcome			0.862
Not yet pregnant (%)	35 (47.3)	67 (48.6)	
Clinical pregnancy (%)	39 (52.7)	71 (51.4)	

In the 2- and 4-mg/day groups, 24 and 64 patients tried to get pregnant naturally after surgery, respectively, and the remaining 50 and 74 patients referred to ART, respectively. At the end of data collection, 39 of 74 patients in the 2-mg/day group obtained clinical pregnancy (31 patients had live births and 8 patients had ongoing pregnancies), 30 patients did not get pregnant throughout the study period, and 5 patients did not become pregnant after spontaneous abortion. Moreover, 71 of 138 patients in the 4-mg/day group were clinically pregnant (49 patients had a live birth and 22 patients had ongoing pregnancies), 65 patients were not pregnant at all, and 2 patients did not become pregnant after spontaneous abortion. The difference in clinical pregnancy rates between the 2- and 4-mg/day estradiol groups was not statistically significant in the primary comparison (52.7% vs. 51.4%, *P* = 0.862). The abortion rates of the 2- and the 4-mg/day estradiol groups were 7.5% and 1.6%, respectively, and the difference was not significant (*P* = 0.087).

Several confounding factors, such as diagnosis classification, preoperative estradiol level, age, BMI, history of hysteroscopic surgery, preoperative menstrual pattern, estradiol treatment regimen, and number of pregnancy-related dilatation and curettage procedures, were adjusted in the two multivariate regression models. Neither the crude comparison nor the two adjusted models showed a significant effect of different doses of estradiol on the clinical pregnancy rates (crude OR = 0.95, 95% CI = 0.54–1.67; adjusted OR of model I = 0.73, 95% CI = 0.39–1.38; adjusted OR of model II = 0.64, 95% CI = 0.32–1.28, **Table 2**).

**Table 2:** Relationship between the daily dose of estradiol valerate and the reproductive outc ome and menstrual improvement in different models.

	Non-adjusted^a^		Adjusted model I^b^		Adjusted model II^c^	
	OR (95% CI)	*P*	OR (95% CI)	*P*	OR (95% CI)	*P*
Reproductive outcome						
2 mg/day	1.0		1.0		1.0	
4 mg/day	0.95 (0.54, 1.67)	0.862	0.73 (0.39, 1.38)	0.337	0.64 (0.32, 1.28)	0.206
Menstrual improvement						
2 mg/day	1.0		1.0		1.0	
4 mg/day	0.93 (0.53, 1.65)	0.814	1.02 (0.55, 1.90)	0.948	1.00 (0.52, 1.93)	0.996

a The non-adjusted model was not adjusted.

b Adjusted model I was adjusted for diagnosis classification and preoperative estradiol level.

c Adjusted model II was adjusted for diagnosis classification, preoperative estradiol level, age, BMI, history of hysteroscopic surgery, preoperative menstrual pattern, estradiol treatment regimen, and number of pregnancy-related dilatation and curettage procedures.

To further confirm the results of the multivariate analysis, PSM was conducted, such that the comparison was in a more homogeneous population. We analyzed the reproductive outcomes in 122 PSM-matched patients (61 patients in the 2-mg/day group and 61 patients in the 4-mg/day group). As shown in **Table 3**, after PSM, no significant differences were found in terms of age, BMI, diagnosis classification, number of pregnancy-related dilatation and curettage procedures, history of hysteroscopic surgery, preoperative menstrual pattern, estradiol treatment regimen, and preoperative estradiol level between the matched groups. Multivariate regression analysis was then performed to identify the independent effect of daily estradiol valerate dose on the clinical pregnancy rate. Considering all the confounding factors mentioned above, the results of the multivariate regression analysis failed to show a significant effect of the daily dose of estradiol on the clinical pregnancy rate (fully adjusted OR = 0.46; 95% CI = 0.20–1.06; *P* = 0.068). The menstrual improvement rates were similar in the matched populations (fully adjusted OR = 0.71; 95% CI = 0.33–1.51; *P* = 0.370) (**Table 4**).

**Table 3:** Characteristics of the propensity-matched groups.

Variables	2 mg/day	4 mg/day	Standard difference	*P*
Age (years)	(61) 34.95 ± 5.32	(61) 34.10 ± 4.35	0.1753	0.335
BMI (kg/m^2^)	(61) 22.09 ± 2.72	(61) 22.46 ± 2.76	0.1357	0.455
Diagnosis classification			0.1089	0.689
Moderate (%)	45 (73.8)	42 (68.9)		
Severe (%)	16 (26.2)	19 (31.1)		
Preoperative estradiol level				0.626
Early follicular stage (%)	9 (14.8)	12 (19.7)	0.1306	
Middle follicular stage (%)	43 (70.5)	38 (62.3)	0.1742	
Late follicular stage (%)	9 (14.8)	11 (18)	0.0886	
History of hysteroscopic surgery			0.1187	0.663
No (%)	49 (80.3)	46 (75.4)		
Yes (%)	12 (19.7)	15 (24.6)		
Preoperative menstrual pattern			0.0000	1.000
Regular (%)	52 (85.2)	52 (85.2)		
Irregular (%)	9 (14.8)	9 (14.8)		
Estradiol treatment regimen			0.0677	0.852
Cyclical (%)	22 (36.1)	24 (39.3)		
Continuous (%)	39 (63.9)	37 (60.7)		
Number of pregnancy-related dilatation and curettage procedures				0.646
None (%)	11 (18)	9 (14.8)	0.0886	
One (%)	21 (34.4)	20 (32.8)	0.0347	
Two (%)	13 (21.3)	19 (31.1)	0.2250	
Three or more (%)	16 (26.2)	13 (21.3)	0.1157	
Outcomes after PSM				
Reproductive outcome			0.2310	0.277
Not yet pregnancy (%)	27 (44.3)	34 (55.7)		
Clinical pregnancy (%)	34 (55.7)	27 (44.3)		
Menstrual improvement			0.1316	0.587
Not improved (%)	27 (44.3)	31 (50.8)		
Improved (%)	34 (55.7)	30 (49.2)		

**Table 4:** Multivariate logistic regression of the propensity-matched groups.

	Non-adjusted^a^		Adjusted model I^b^		Adjusted model II^c^	
	OR (95% CI)	*P*	OR (95% CI)	*P*	OR (95% CI)	*P*
Reproductive outcome						
2 mg/day	1.0		1.0		1.0	
4 mg/day	0.63 (0.31, 1.29)	0.206	0.45 (0.20, 1.01)	0.052	0.46 (0.20, 1.06)	0.068
Menstrual improvement						
2 mg/day	1.0		1.0		1.0	
4 mg/day	0.77 (0.38, 1.57)	0.469	0.69 (0.32, 1.46)	0.328	0.71 (0.33, 1.51)	0.370

a The non-adjusted model was not adjusted.

b Adjusted model I was adjusted for age, number of pregnancy-related dilatation and curettage procedures, and preoperative estradiol level.

c Adjusted model II was adjusted for age, number of pregnancy-related dilatation and curettage procedures, preoperative estradiol level, history of hysteroscopic surgery, and preoperative menstrual pattern.

## Discussion

In this study, we explored the effect of 2- and 4-mg/day doses of estradiol valerate on the prognosis of IUAs, which could provide evidence for clinical practice. Our findings indicated that the daily dose of estradiol valerate was not correlated with the clinical pregnancy rate of IUAs. Moreover, no significant difference was found between the effects of the 2- and the 4-mg/day dose of estradiol valerate on the improvement of menstrual volume.

Hysteroscopic adhesiolysis is the primary treatment for IUAs. However, the re-adhesion rate in patients with moderate to severe IUAs is as high as 30%–62.5% (19, 20). Estradiol therapy is one of the most important adjuvant measures to promote endometrial hyperplasia; thus, the residual endometrium can quickly cover the surgical wound to prevent the formation of postoperative adhesions as effectively as possible. A net meta-analysis suggested that, compared with physical barriers, biological gel, stem cells, and an amniotic membrane, estradiol was the most effective adjuvant measure in preventing the recurrence of postoperative adhesions (21). However, the dose of estradiol is not uniform in clinical practice. Within our search of existing literature, no studies have compared 2 with 4 mg of estradiol daily for the prognosis of IUAs.

Some studies have shown no difference in the improvement of prognosis between the lower and higher doses of estradiol after surgery. A randomized controlled trial conducted in China compared the effect of different estradiol doses (2 vs. 6 mg/day) on inhibiting postoperative endometrial fibrosis. Their findings suggested that a lower dose of estradiol appeared to be more beneficial for the treatment of IUAs (8). Regretfully, although this study controlled for potential confounding factors by randomization, the pregnancy outcome, which was regarded as the more important indicator of prognosis in patients with IUAs, was not compared in this study. In 2019, a retrospective study involving 176 women with moderate to severe IUAs assessed the changes in the American Fertility Society score, pregnancy rate, and abortion rate between the 4- and 10-mg/day estradiol groups, and the results supported the use of a lower dose of estradiol after hysteroscopic adhesiolysis (10). Animal experiments have also confirmed that a moderate dose of estradiol supplementation is more helpful in endometrial repair than a high dose of estradiol supplementation (17, 22). Therefore, a lower estradiol dose is recommended. In addition, previous studies showed that the use of 2 mg/day of estradiol after hysteroscopic surgery, such as hysteroscopic septal resection and hysteroscopic myomectomy, was also conducive to reducing the incidence of IUAs (23, 24). In other studies, beneficial effects on endometrial repair were also observed when 4 mg/day of estradiol valerate was used (1, 25). Nonetheless, these studies did not include a control group to compare the effects or side effects of different doses of estradiol on endometrial regeneration. The dose of 2 mg/day of estradiol valerate was the lowest dose that has been reported as the adjuvant treatment for IUAs within the scope of our literature review.

Thus far, the mechanisms underlying the effect of estradiol on endometrial repair remain unclear. It is universally acknowledged that the number and functional status of ERs determine the level of local endometrial estradiol and the biological effects. Studies have shown that compared with the IUAs and normal endometrium, ERs are expressed at higher levels in the endometrium of patients with IUAs; however, the function of ERs in IUAs is defective. For example, the binding of estradiol and receptor is reduced, which leads to poor angiogenesis and endometrial regeneration (13). On the contrary, according to the pharmacokinetic characteristics of estradiol, the serum concentration of estradiol increased with an increase in oral dose, within a single oral dose of 1–4 mg of estradiol. However, the increase was not obvious when the dose exceeded 8 mg. Furthermore, the results of an animal experiment suggested that high-dose estradiol may promote endometrial fibrosis and IUA formation by upregulating some cytokines, such as TGF-β and bFGF (15). Therefore, we believe that the use of a high dose of estradiol valerate after surgery and the formation of an excessive estradiol environment are not conducive to postoperative wound repair in the endometrium.

We considered two advantages of our study. One advantage of the present study was that scientific statistical methods were applied. Although baseline imbalance was intrinsically involved in the retrospective study, to reduce the risk of bias, confounding factors were adjusted in the regression model to evaluate the independent effect of the exposure factor on the outcomes. We further applied PSM to balance the difference in indicators between the two groups, which makes the results more reliable. Another advantage was that, within our search scope, the sample size of our study was the largest among similar studies. A larger sample size is beneficial for reducing sampling errors during statistics.

This study has some limitations: First, it is a retrospective study with some intrinsic limitations such as potential selection bias despite all the efforts to control it (26). Therefore, well-designed randomized controlled trials are expected in the future. Second, the *P*-value (*P* = 0.068) in adjusted model II after PSM seems to have a trend of significance, which may be associated with the relatively low statistical power of this study (61.6%). Given that the insufficient sample size leads to insufficient statistical power to detect a slight difference, the results should be interpreted with caution as they need to be confirmed or disproved in prospective studies. In addition, we calculated the sample size of the prospective study, which should reach 602 (1:1) at the power of 0.80. Hopefully, a well-designed randomized controlled trial with a sufficient sample size will be conducted in the future. Third, the data regarding IUA recurrence rate were lacking in this paper due to the following reasons: First, the patients who had totally recovered stopped undergoing hysteroscopic examination, and therefore, we do not know if the IUAs recurred or not; second, there were some patients whose uterine cavity did not completely recover at the end of the follow-up period, and thus, we do not know whether the IUAs recurred or not. It is hoped that the relevant data can be collected in the future. Finally, our study was conducted in Northwest China, and caution should be taken when extrapolating conclusions to other populations.

In conclusion, our results indicated no significant difference in the effect on improving the reproductive outcomes or menstrual volume between the 2- and 4-mg/day estradiol valerate groups. Therefore, it is recommended that a lower effective dose (2 mg/day) of estradiol valerate be administered as adjuvant therapy after hysteroscopic adhesiolysis to reduce estrogen-related adverse effects.

## Data Availability Statement

The raw data supporting the conclusions of this article will be made available by the authors, without undue reservation.

## Author Contributions

D-EQ collected the data and completed the manuscript. W-LZ designed the study, critically reviewed the manuscript, and performed the hysteroscopic surgeries. As resident doctors, JL, FY, YM, and Y-FT were in charge of the treatment process of every patient. X-FX provided the treatment strategy of estradiol and performed the final review. X-HW supervised the study and made the final review of the paper. All authors contributed to the article and approved the submitted version.

## Conflict of Interest

The authors declare that the research was conducted in the absence of any commercial or financial relationships that could be construed as a potential conflict of interest.

## Publisher’s Note

All claims expressed in this article are solely those of the authors and do not necessarily represent those of their affiliated organizations, or those of the publisher, the editors and the reviewers. Any product that may be evaluated in this article, or claim that may be made by its manufacturer, is not guaranteed or endorsed by the publisher.
